# Assessing age-related risks of gastrointestinal pathologies: a comparative study of gastroscopy outcomes across decades

**DOI:** 10.1177/17562848241290446

**Published:** 2024-10-28

**Authors:** Lac Nguyen, Noora Räsänen, Filippa Berggren, Michiel A. van Nieuwenhoven

**Affiliations:** Division of Gastroenterology, Department of Internal Medicine, and University Health Care Research Unit, Faculty of Medicine and Health, Örebro University, Örebro, Sweden; Division of Gastroenterology, Department of Internal Medicine, and University Health Care Research Unit, Faculty of Medicine and Health, Örebro University, Örebro, Sweden; Division of Gastroenterology, Department of Internal Medicine, and University Health Care Research Unit, Faculty of Medicine and Health, Örebro University, Örebro, Sweden; Division of Gastroenterology, Department of Internal Medicine, Faculty of Medicine and Health, Örebro University, Södra Grev Rosengatan, Örebro SE-70182, Sweden

**Keywords:** cancer, dyspepsia, esophagogastroduodenoscopy, gastroscopy, guidelines

## Abstract

**Background:**

Esophagogastroduodenoscopy (EGD) is the gold standard method for diagnosing upper gastrointestinal (GI) pathology. Swedish guidelines recommend patients over 50 years with new-onset dyspeptic symptoms undergo direct gastroscopy to rule out malignancy. However, the incidence of dysplasia or cancer in patients aged 61–70 years remains unclear.

**Objectives:**

To investigate the referral factors and endoscopic findings in patients aged 61–70 years and compare the result with age groups 51–60 and 41–50 years from our previous studies to establish whether there is an age cutoff for upper GI cancer risk.

**Design:**

A retrospective observational study was conducted to evaluate EGD referrals and outcomes in patients aged 61–70 years.

**Methods:**

We analyzed EGD referrals for patients aged 61–70 years within Region Örebro County from January 2019–April 2020 to January 2022–2023. Clinical data, including symptoms, medications, and laboratory results, were collected from medical records. Statistical analysis, including odds ratios (OR) and positive predictive values (PPV), was conducted to evaluate pathological outcomes based on referral factors.

**Results:**

A total of 1003 referrals were analyzed. Statistically significant differences in pathological findings were observed between the 41–50 years reference group and the older groups (51–60 years: OR 2.08, *p* < 0.001; 61–70 years: OR 3.05, *p* < 0.001). However, no statistically significant difference in cancer incidence was found between the age groups.

**Conclusion:**

The most common pathological findings were benign, including hiatal hernia, gastroesophageal reflux disease/esophagitis, or gastritis. The incidence of cancer was low in all three groups. These results suggest that the “test-and-treat” strategy, currently recommended for patients under 50 years, may be appropriate for patients aged 51–70 years as well.

**Trial registration:**

NCT04585516.

## Introduction

Upper gastrointestinal (GI) tract malignancy is an important cause of mortality, although regional differences exist. Esophagogastroduodenoscopy (EGD) is the gold standard method for diagnosing upper GI pathology. Although this is the preferred method, overuse is resource-demanding and unpleasant for the patient. One-third of all EGDs performed resulted in no pathological findings. The most common pathological findings comprise benign pathologies including hiatal hernia, esophagitis, or gastritis,^1^ with gastroesophageal cancers being rare (<0.4%).^2^

Older age is considered a risk factor for gastric cancer and esophageal cancer, which are the third and sixth leading causes of cancer mortality worldwide, respectively. These upper GI cancers are often diagnosed in advanced stages due to the lack of early clinical symptoms.^3–5^

Overall, 40% of patients undergoing EGD have dyspepsia.^2^ The global prevalence of uninvestigated dyspepsia is 21%.^6^ The most common type of dyspepsia is functional dyspepsia, where no structural disease can be identified during an EGD.^7^ In the Rome IV criteria, functional dyspepsia is defined as a complex of symptoms, with at least one of the following symptoms: postprandial fullness, early satiation, epigastric pain, or burning, presented in the last 3 months and onset at least 6 months before diagnosis.^8^ The main risk factors for uninvestigated dyspepsia include female gender, non-steroid anti-inflammatory drug (NSAID) use, and *Helicobacter pylori* infection.^6^

According to Swedish guidelines, patients >50 years, presenting with new-onset dyspeptic symptoms, are recommended to undergo direct EGD to exclude cancer.^9^ Patients with alarm symptoms such as anemia, hematemesis, melena, positive fecal hemoglobin (f-Hb), unintended weight loss, persistent vomiting, and dysphagia should be referred for urgent EGD. British guidelines recommend direct EGD in patients aged 55 years or older with dyspepsia and weight loss or treatment-resistant dyspepsia.^10^ However, the American College of Gastroenterology and the Canadian Association of Gastroenterology suggest a different age cutoff, over 60 years. Patients with only dyspeptic symptoms below these age limits should undergo “test-and-treat,” which involves *H. pylori* testing and eradication treatment if positive. Patients with negative tests should be treated with a trial of proton pump inhibitor (PPI) treatment.^11^ Medications such as NSAIDs or acetylsalicylic acid (ASA) should also be discontinued.^9^ Furthermore, *H. pylori* eradication may decrease the incidence of gastric cancer.^12^

Over 6000 EGDs are performed annually in Region Örebro County (RÖC). Our previous studies show that there is no statistically significant difference in the incidence of dysplasia/cancer in patients aged 51–60 years compared to 41–50 years when analyzing EGD referrals and outcomes. However, these previous studies did not include patients over 60 years, which may be of interest when comparing the results of the Swedish with American/Canadian guidelines.^13^ In addition, a study by de Jong et al.,^14^ aiming to detect >80% of malignancies, suggests setting the age threshold for detecting over 80% of malignancies at above 55 years for populations in Europe and North America.

## Aim

This study aimed to investigate the referral factors and EGD findings in an older population, 61–70 years in the RÖC as well as to investigate differences in pathological findings, especially cancer, between our study group and younger age groups 41–50 and 51–60 years from our previous studies. All age groups were analyzed with consistent methods across all studies.

## Materials and methods

### Study design

We conducted a retrospective observational study. We analyzed EGD referrals for patients aged 61–70 years within RÖC during two distinct periods: from January 1, 2019 to April 1, 2020, and from January 1, 2022 to January 15, 2023. EGDs performed during the COVID-19 pandemic were excluded to prevent selection bias, as only urgent procedures were conducted during this time. There were no protocol changes in EGD or referral practices between these periods. The age groups 41–50, 51–60, and 61–70 years were selected based on the significant variations in GI pathology prevalence across these decades. In addition, these age groups align with both national and international guidelines for upper GI endoscopy, which often set different thresholds for endoscopic investigation based on age.

The reporting of this study conforms to the Strengthening the Reporting of Observational Studies in Epidemiology statement.^15^

### Data collection

Patients were selected consecutively based on the date of investigation and manually extracted from a database of EGDs performed in RÖC. We collected clinical data for each patient, including overall symptoms, alarm symptoms, and symptom duration. Demographic data included prior GI diseases, past EGDs, medications, and laboratory results. In cases where the referral text was incomplete or ambiguous, further review of medical records was conducted. Data regarding the concomitant use of NSAIDs, ASA, and PPI that were either mentioned in the referral text or registered in the medical records within 3 months of examination were also collected. Laboratory results for *H. pylori* testing and f-Hb were registered if performed within 12 and 3 months, respectively, prior to the examination date.

Pathological findings comprised of hiatal hernia, gastroesophageal reflux disease (GERD)/esophagitis, gastritis/duodenitis, ventriculi ulcer, duodenal ulcer, healed ulcer, esophageal varices, celiac disease, strictures, dysplasia, and cancer. Barrett’s esophagus was included in the group GERD/esophagitis. Polyps without dysplasia and rare benign upper GI conditions like gastric antral vascular ectasia were not recorded. Pathology reports were reviewed if necessary.

### Statistical analysis

Statistical analysis was conducted using IBM SPSS Statistics version 28.0.0.0. Positive predictive values (PPVs) were calculated by dividing the number of pathological EGDs with a referral factor by the total counts of EGDs with that factor. Odds ratios (ORs) were calculated by comparing the odds of pathological outcomes in EGDs with a referral factor against those without. For categorical variables, Pearson’s Chi-squared test or Fisher’s exact test was applied if any parameter had an expected frequency less than 5. A receiver operating characteristic (ROC) curve regarding the relationship between age and upper GI cancer for the age group 51–70 years was constructed. Differences in pathological findings and rates of dysplasia/cancer were analyzed between age groups, with either the 41–50 or 51–60 years cohort serving as a reference. A *p*-value of less than 0.05 was considered statistically significant.

## Results

**Figure 1.**
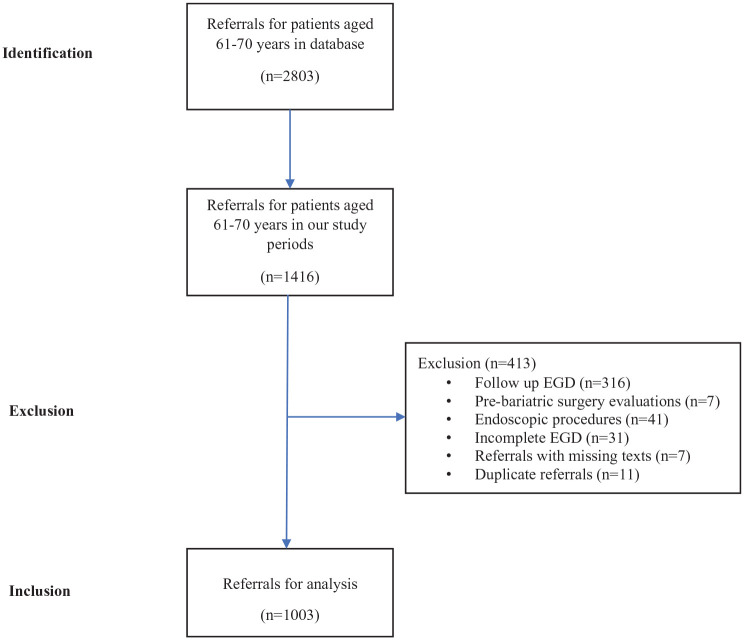
Flow chart of data collection and referrals inclusion.

A total of 2803 EGD referrals in patients 61–70 years were found in the database. From this group, 1416 referrals from two distinct study periods, January 2019–April 2020 and January 2022–2023 were identified. Following a systematic review, 413 referrals were excluded for reasons illustrated in Figure 1. Exclusions included referrals for endoscopic treatment of esophageal strictures and varices, follow-ups on previously diagnosed ulcer diseases, and pre-bariatric surgery evaluations. In addition, referrals with missing texts, incomplete or interrupted gastroscopy examinations, and duplicate referrals were excluded. However, referrals for conditions such as iron deficiency anemia, GI bleeding, celiac disease, and esophageal varices were included. Incomplete EGDs were primarily due to poor patient tolerance, technical difficulties, or obstructive lesions such as strictures or tumors. These cases were included in the analysis if they provided sufficient diagnostic information regarding the presence or absence of malignancy or other significant pathology. Ultimately, 1003 EGD referrals were included in the final analysis.

In our study, 72.7% (*n* = 729) of the referrals yielded pathological EGD findings. There was a higher proportion of women (54.8%) compared to men (45.2%), with women predominating particularly in the group without pathological findings (65.0%), as shown in Table 1. Women were less likely to have pathological findings in their EGDs (OR 0.56), compared to men (OR 1.78). In 19 referrals (1.9%), no symptoms were reported; these typically involved EGD for conditions like celiac disease or vitamin B12 deficiency, prompted by abnormal laboratory results such as elevated levels of anti-tissue transglutaminase antibodies or reduced vitamin B12 levels. The presence of alarm features was nearly identical between the non-pathological (67.5%), and the pathological groups (66.9%), with no alarm features showing a statistically significant association with pathological findings.

**Table 1.** Frequencies, PPVs, ORs, and *p*-values for patient characteristics and symptoms that were mentioned in the referrals in relation to pathological findings and non-pathological findings in the endoscopic outcomes.

Patient characteristics	Pathological EGD		PPV, %	OR (95% CI)	*p* Value
	Yes (*n* = 729), *n* (%)	No (*n* = 274), *n* (%)			
**Patient characteristics**					
Women	372 (51.0)	178 (65.0)	67.6	0.56 (0.42–0.75)	<0.001
Men	357 (49.0)	96 (35.0)	78.8	1.78 (1.34–2.37)	<0.001
Mean age (years)	64.5	64.2			
**Symptom duration (months)**					
<1	135 (18.5)	53 (19.3)	71.8	0.95 (0.67–1.35)	0.766
1–6	152 (20.9)	73 (26.6)	67.6	0.73 (0.53–1.0)	0.050
>6	178 (24.4)	64 (23.4)	73.6	1.06 (0.76–1.47)	0.727
Similar symptoms earlier	179 (24.6)	73 (26.6)	71.0	0.90 (0.65–1.23)	0.497
No information	254 (34.8)	80 (29.2)	76.0	1.3 (0.96–1.75)	0.091
**Symptom**					
Symptom mentioned in referrals	714 (98.0)	270 (98.5)	72.6	0.71 (0.23–2.14)	0.536
Heartburn/regurgitation/retrosternal chest pain	190 (26.1)	52 (19.7)	80.3	1.44 (1.02–2.02)	0.037
Other reflux symptoms (cough, sore throat, globus, hiccups)	73 (10.0)	31 (11.1)	70.2	0.87 (0.60–1.36)	0.547
Epigastric pain/discomfort/burning	268 (36.8)	113 (41.2)	70.3	0.83 (0.62–1.10)	0.193
Early satiation	28 (3.8)	18 (6.6)	60.9	0.57 (0.31–1.05)	0.066
Other dyspeptic symptoms (abdominal bloating)	56 (7.7)	34 (12.4)	62.2	0.59 (0.37–0.92)	0.02
Occasional nausea	63 (8.6)	41 (15.0)	60.6	0.54 (0.35–0.82)	0.003
Occasional vomiting	49 (6.7)	19 (6.9)	72.1	0.97 (0.56–1.67)	0.905
Chest pain	17 (2.3)	8 (2.9)	68	0.79 (0.34–1.86)	0.595
Abdominal pain	87 (11.9)	47 (17.2)	64.9	0.66 (0.45–0.96)	0.030
Diarrhea/loose stools	61 (8.4)	22 (8.0)	73.5	1.05 (0.63–1.74)	0.862
**Alarm features**					
Alarm features mentioned	488 (66.9)	185 (67.5)	72.5	0.97 (0.72–1.31)	0.862
Anemia	196 (26.9)	62 (22.6)	76.0	1.26 (0.91–1.74)	0.169
Melena or hematochezia	87 (11.9)	27 (9.9)	76.3	1.24 (0.79–1.96)	0.355
Hematemesis	40 (5.5)	11 (4.0)	78.4	1.34 (0.70–2.75)	0.344
Positive fecal hemoglobin	110 (15.1)	59 (21.5)	65.1	0.65 (0.40–1.07)	0.088
Unintentional weight loss	94 (12.9)	45 (16.4)	67.6	0.75 (0.51–1.11)	0.149
Loss of appetite	48 (6.6)	18 (6.6)	72.7	1.0 (0.57–1.76)	0.993
Dysphagia or odynophagia	122 (16.7)	37 (13.5)	76.7	1.29 (0.87–1.92)	0.212
Persistent vomiting or nausea	66 (9.1)	26 (9.5)	71.7	0.95 (0.59–1.53)	0.831
Palpable mass in the abdomen	0	2	0		0.074
Jaundice	0	0			

PPVs were determined by dividing the number of pathological EGDs with a referral factor by the total counts of EGDs with that factor. ORs were calculated by dividing the odds of pathological EGDs with a referral factor by the odds of pathological EGD without that factor. For categorical variables, Pearson’s Chi-squared test was applied, or Fisher’s exact test if any parameter had an expected frequency less than 5.

CI, confidence interval; EGD, esophagogastroduodenoscopy; OR, odds ratio; PPV, positive predictive value.

Diagnostics and treatments prior to EGD are presented in Table 2. The OR for pathological versus non-pathological EGD outcomes in EGD referrals according to the standardized course of care (SCC) and the fast-track pathway for suspected upper GI cancer were identical (OR 1.0). Patients who had undergone bariatric surgery showed a lower association with pathological findings (OR 0.38). However, patients with previously performed EGD or with known GI diseases of the esophagus, stomach, or liver did not show a statistically significant association with a pathological outcome; OR 1.02 and OR 1.07, respectively. Treatments involving NSAIDs or ASA, as mentioned in the referrals or documented in medical records within 3 months prior to the EGD, were significantly associated with pathological findings. *Helicobacter pylori* testing, whether mentioned in the referral or documented in records up to 12 months before the examination, was conducted in 17.0% of cases, with 18.1% testing positive. However, a positive *H. pylori* test did not correlate significantly with pathological gastroscopy findings.

**Table 2.** Frequencies, PPVs, ORs, and *p*-values for referral indications, diagnostics, and treatment before EGD in relation to pathological findings and non-pathological findings in the EGD outcomes.

	Pathological EGD		PPV, %	OR (95% CI)	*p* Value
	Yes (*n* = 729), *n* (%)	No (*n* = 2*7*4), *n* (%)			
**Indications**					
SCC referral	74 (10.2)	28 (10.2)	72.5	1.0 (0.63–1.58)	0.975
Celiac disease referral	9 (1.2)	1 (0.4)	90	3.4 (0.43–27.10)	0.217
Iron-deficiency anemia	79 (10.8)	25 (9.1)	75.2	1.21 (0.75–1.94)	0.428
Varices	34 (4.7)	5 (1.8)	87.2	2.6 (1.02–6.80)	0.038
**Diagnostics before gastroscopy**					
Previous bariatric surgery	21 (2.9)	20 (7.3)	51.2	0.38 (0.20–0.71)	0.002
Previous gastroscopy	264 (36.2)	98 (35.8)	72.9	1.02 (0.76–1.36)	0.895
Previous gastrointestinal diagnosis	291 (39.9)	105 (38.3)	73.5	1.07 (0.80–1.42)	0.645
*H. pylori* testing	124 (17.0)	47 (17.2)	72.5	0.99 (0.69–1.43)	0.957
*H. pylori* positive	20 (2.7)	11 (4.0)	64.5	0.63 (0.26–1.44)	0.270
Fecal hemoglobin testing	213 (29.2)	95 (34.7)	69.2	0.78 (0.58–1.05)	0.095
**Treatments before gastroscopy**					
PPI	433 (59.4)	146 (53.3)	74.8	1.28 (0.97–1.70)	0.081
NSAIDs or ASA	227 (31.1)	66 (24.1)	77.5	1.43 (1.04–1.96)	0.029

PPVs were determined by dividing the number of pathological EGDs with a referral factor by the total counts of EGDs with that factor. OR was calculated by dividing the odds of pathological EGDs with a referral factor by the odds of pathological EGD without that factor. For categorical variables, Pearson’s Chi-squared test or Fisher’s exact test if any parameter had an expected frequency less than 5.

ASA, acetylsalicylic acid; CI, confidence interval; EGD, esophagogastroduodenoscopy; NSAIDs, non-steroidal anti-inflammatory drugs; OR, odds ratio; PPI, proton-pump inhibitor; PPV, positive predictive values; SCC, standardized course of care.

**Figure 2.**
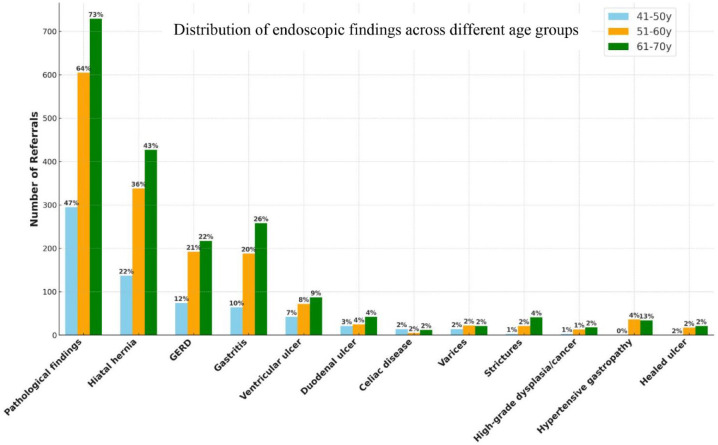
Number of referrals and their endoscopic findings in the age groups 41–50, 51–60, and 61–70 years.

We included previously collected data from the age groups 41–50 and 51–60 years (Figure 2).^13^ The number of gastroscopies in the age group, 41–50 years (*n* = 633) was lower compared to 51–60 years (*n* = 939) and 61–70 years (*n* = 1003). The incidence of pathological findings was lowest in the group 41–50 years group (46.6%).

The most common endoscopic findings across all age groups were hiatal hernia, GERD/esophagitis, gastritis, and ventricular ulcer. Despite having the fewest referrals, the 41–50 years group exhibited the highest frequency of celiac disease relative to the other groups. In total, our study identified 18 cases of dysplasia or cancer, which included 1 case of high-grade dysplasia, 12 cases of gastric cancer, and 5 cases of esophageal cancer. In addition, 5 cases of low-grade dysplasia were noted but were not classified under malignancies.

For comparative analysis, the age groups 41–50 and 51–60 years were used as reference groups (Table 3). There were statistically significant differences in pathological findings between the 41–50 years and the older groups 51–60 years (OR 2.08, *p* < 0.001) and 61–70 years (OR 3.05, *p* < 0.001). When using the 51–60 years group as a reference, the comparison with the 61–70 years group also revealed a statistically significant difference (OR 1.47, *p* < 0.001). The frequencies of dysplasia and cancer were low across all three groups, and no statistically significant differences were observed among them.

**Table 3.** Comparison of pathological findings and dysplasia/cancer between the age groups.

Age groups (years)	Pathological findings		Cancer	
	OR (95% CI)	*p* Value	OR (95% CI)	*p* Value
41–50	Ref	Ref	Ref	Ref
51–60	2.08 (1.60–2.55)	<0.001	1.76 (0.63–4.97)	0.283
61–70	1.47 (1.21–1.78)	<0.001	2.30 (0.85–6.21)	0.102
51–60	Ref	Ref	Ref	Ref
61–70	1.47 (1.21–1.78)	<0.001	1.30 (0.63–2.67)	0.472

CI, confidence interval; OR, odds ratio.

**Figure 3.**
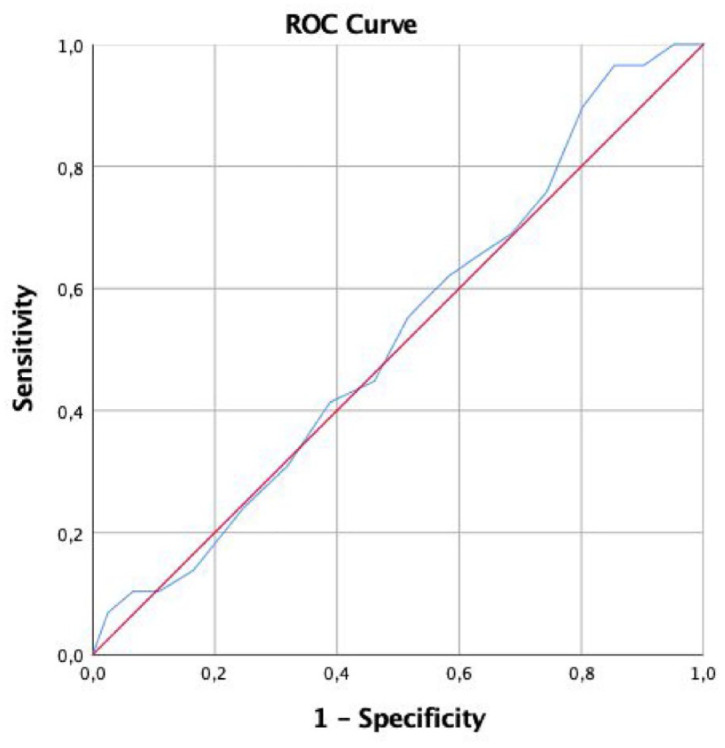
ROC curve examining the predictive value of age for cancer diagnosis among patients aged 51–70 years.

A ROC curve analysis examining the predictive value of age for cancer diagnosis among patients aged 51–70 years indicated an area under the curve of 0.524, suggesting minimal discriminative power (Figure 3). The result, with a standard error of 0.050 and a non-significant *p*-value of 0.661, supports that age alone is a weak predictor of cancer in this age group. The 95% CI ranged from 0.426 to 0.622, further confirming the limited utility of age as a diagnostic predictor in this cohort.

## Discussion

### Main outcomes of this study

Our study evaluated patients undergoing EGD in RÖC. We observed no statistically significant differences in malignancy rates across the age groups of 41–50, 51–60, and 61–70 years. The ROC analysis indicated that age alone is a poor predictor of cancer.

While age itself is a significant risk factor for GI pathologies, it is important to consider other aging-related factors. Comorbidities, prolonged use of medications, and lifestyle factors, including smoking, alcohol consumption, and diet, also play important roles. Notably, chronic NSAID use, along with persistent *H. pylori* infection, which is more prevalent in older adults, remains a critical factor in the development of peptic ulcer disease, gastritis, and gastric cancer.

The age group of 61–70 years exhibited more pathological but benign findings than the younger cohorts, such as hiatal hernia, GERD/esophagitis, and gastritis, aligning with previous studies.^1,16^ These outcomes suggest that, contrary to Swedish guidelines, patients over 50 years could benefit from the “test and treat” strategy involving *H. pylori* testing and eradication before proceeding with an invasive EGD. Notably, peptic ulcers were observed across all three age groups, primarily due to *H. pylori* infection or NSAID use as the main risk factors.^17^ Preventing peptic ulcer disease is essential to avoid severe complications like upper GI bleeding and perforation. Alarm symptoms such as hematemesis and melena typically indicate upper GI bleeding and peptic ulcer disease is frequently associated with nonspecific symptoms such as epigastric pain, abdominal bloating, or early satiety, which are also associated with dyspepsia.^18^

We chose not to further break down “Pathological findings” in Table 1 into categories like benign pathological findings and malignancies due to detailed analyses from a previous study.^19^ That study showed that symptoms such as dysphagia, nausea/emesis, and hematemesis had no predictive value for upper GI cancer, whereas unintentional weight loss, GI bleeding, early satiety, and radiological findings suggestive of malignancies were significant predictors. Age above 50 years, while showing an increase in OR, did not reach statistical significance. In addition, other risk factors such as smoking, alcohol use, and familial risk of cancer were also analyzed. Male sex was identified as an independent risk factor, former smoking posed a moderate risk, and alcohol consumption showed no significant association with upper GI cancer. However, due to incomplete or missing data on smoking and alcohol use in patient records, these variables were not included in the primary analysis of the current study.

According to the current Swedish Guidelines for the management of dyspepsia, the primary causes of peptic ulcer disease are addressed by discontinuation of NSAIDs and applying a noninvasive “test-and-treat” strategy in patients under 50 years, before conducting an EGD. *Helicobacter pylori* eradication has been effective in treating peptic ulcer disease,^20^ with patients testing negative for *H. pylori* being treated with PPIs, which are also effective for the majority of benign upper GI pathologies.

In our study, only 17% of the 61–70 age group were tested for *H. pylori* prior to EGD. Notably, this proportion was similar to that observed in the age group of 18–50 years,^13^ despite recommendations specifically targeting patients under 50 years with uninvestigated dyspepsia for such testing. This suggests a uniform approach to managing dyspepsia across all ages, with poor adherence to the national guidelines. As a result, pathologies associated with *H. pylori* may be underdiagnosed, and consequently, undertreated. Gupta et al.^21^ have reported a deficiency in *H. pylori* testing prior to EGD in the United States, with 75% of gastroscopies not adhering to ACG/CAG guidelines.

### The association between aging and gastroesophageal malignancies

Previous studies have examined the correlation between aging and the incidence of gastroesophageal malignancies. A meta-analysis from de Jong et al. showed that limiting upper GI endoscopy to patients >55 years of age will detect 85% of malignancies. In geographical areas with a lower prevalence of upper GI malignancy, a limit of >60 years of age may be justified, particularly in patients who present with dyspepsia in the absence of alarm symptoms.^14^ Our data show that the highest incidence of malignancy is found in the 61–70-year age group at 1.8%, compared to 1.4% in the 51–60-year group and 0.8% in the 41–50-year group. These rates in the 61–70-year group closely mirror earlier findings, which reported a 1.9% incidence in the 65–69-year age bracket.^22^ Although these figures suggest an increase in malignancy with age, no statistical significance was found across the age groups. This indicates that a noninvasive “test-and-treat” approach might be more appropriate due to the relatively low incidence of malignancies, even as the frequency of benign pathologies increases with age.

### Swedish SCC for upper GI cancer

In 2016, Sweden introduced the SCC for upper GI cancer, a fast-track pathway designed to minimize delays in diagnosis and treatment.^23^ This pathway recommends immediate EGD referral for upper GI alarm symptoms: conditions like recent-onset dysphagia, persistent emesis, or early satiety over 3 weeks, severe unintentional weight loss, GI bleeding, iron deficiency anemia, or radiological indicators of esophageal or gastric cancer. A recent study highlighted that upper GI cancer was detected in 6.2% of SCC referrals, significantly higher than the cancer yield in our study. Notably, only a few SCC entry criteria, including early satiety, radiological findings, and unintentional weight loss, were significantly associated with an elevated risk of upper GI cancer.^19^

### Guidelines and alarm symptoms in EGD

Both Swedish and American guidelines for the management of uninvestigated dyspepsia mandate prompt gastroscopy for patients presenting with alarm symptoms, to exclude serious conditions such as upper GI bleeding or malignancy.^9,11^ Contrary to these guidelines, our study found no statistical correlation between alarm symptoms and pathological findings in gastroscopy, with ORs remaining low across all alarm symptoms. This lack of correlation is consistent with findings in younger demographics (18–50 years)^13^ and is supported by studies indicating that alarm symptoms are unreliable predictors of upper GI malignancies.^24,25^

Our dataset included a higher proportion of women referred for EGD than men. Previous research has identified female gender as a risk factor for dyspepsia.^6^ Despite this, women exhibited fewer pathological findings than men. This pattern aligns with findings from our earlier studies involving the 41–50- and 51–60-year age groups, suggesting that women may either be more susceptible to dyspeptic symptoms or more proactive in seeking medical care.^13^ Conversely, male gender has been identified as an independent predictor of significant endoscopic findings such as ulcers or malignancy, particularly in elderly patients.^22^

### Strengths and limitations

The study’s limitations include its restricted geographical scope to a single Swedish county, potentially affecting the broader applicability of its findings due to local differences in guidelines, healthcare access, and demographics. The retrospective design based on existing medical records and gastroscopy referrals may have led to data gaps and misclassification errors. Different investigators analyzing age-specific data could introduce interpretation bias.

The COVID-19 pandemic significantly impacted healthcare delivery, leading to delays in diagnostic procedures. It may be possible that the pandemic may have contributed to a higher rate of pathological findings due to delayed investigation.

While our study benefits from a large overall sample size, the relatively low incidence of upper GI cancers limits the power to detect significant differences in cancer rates across age groups. Due to logistical constraints, we were unable to increase the sample size. However, our findings are consistent with similar studies, suggesting that the sample size is adequate for the scope of this research. Future studies with larger cohorts or multi-center collaborations may be necessary to confirm these findings.

## Conclusion

Our study identified significantly more pathological findings in the 51- to 60- and 61- to 70-year age groups compared to the 41- to 50-year group. The most common pathological findings across these groups were benign conditions, including hiatal hernia, GERD/esophagitis, and gastritis. The incidence of cancer was low across all age groups, with no statistically significant differences observed among them. These results suggest that the “test-and-treat” strategy, currently recommended for patients under 50 years, may be extended to individuals aged 51–70. The findings advocate for noninvasive approaches to managing dyspepsia in the community, reserving EGD for those at high risk of malignancy, especially when the SCC for upper GI cancer is applied. A nationwide study would be valuable to reevaluate the appropriateness of the current age cutoff (>50 years) in the Swedish guidelines for direct EGD in patients presenting with dyspeptic symptoms.

## Declarations

**Ethics approval and consent to participate:** This study was approved by the Regional Ethics Committee (Dnr. 2023-03776-02) in Uppsala.

**Consent for publication:** Not applicable.

**Availability of data and materials:** The data are not available due to patient privacy.

## References

[1] Barret M Chaussade S Boustiere C , et al. Diagnostic yield of esophagogastroduodenoscopy in France. Clin Res Hepatol Gastroenterol 2021; 45(4): 101540. 33036954 10.1016/j.clinre.2020.08.015

[2] Nasseri-Moghaddam S Mousavian AH Kasaeian A , et al. What is the prevalence of clinically significant endoscopic findings in subjects with dyspepsia? Updated systematic review and meta-analysis. Clin Gastroenterol Hepatol 2023; 21(7): 1739–1749.e2. 10.1016/j.cgh.2022.05.041 35738355

[3] Smyth EC Nilsson M Grabsch HI , et al. Gastric cancer. Lancet 2020; 396(10251): 635–648. 32861308 10.1016/S0140-6736(20)31288-5

[4] Bray F Ferlay J Soerjomataram I , et al. Global cancer statistics 2018: GLOBOCAN estimates of incidence and mortality worldwide for 36 cancers in 185 countries. CA Cancer J Clin 2018; 68(6): 394–424. 30207593 10.3322/caac.21492

[5] Huang FL Yu SJ. Esophageal cancer: risk factors, genetic association, and treatment. Asian J Surg 2018; 41(3): 210–215. 27986415 10.1016/j.asjsur.2016.10.005

[6] Ford AC Marwaha A Sood R , et al. Global prevalence of, and risk factors for, uninvestigated dyspepsia: a meta-analysis. Gut 2015; 64(7): 1049–1057. 25147201 10.1136/gutjnl-2014-307843

[7] Ford AC Marwaha A Lim A , et al. What is the prevalence of clinically significant endoscopic findings in subjects with dyspepsia? Systematic review and meta-analysis. Clin Gastroenterol Hepatol 2010; 8(10): 830–837, 837.e1–2. 20541625 10.1016/j.cgh.2010.05.031

[8] Stanghellini V Chan FK Hasler WL , et al. Gastroduodenal disorders. Gastroenterology 2016; 150(6): 1380–1392. 27147122 10.1053/j.gastro.2016.02.011

[9] Agréus L Simrén M. Nya riktlinjer för handläggning av dyspepsi, *H pylori* och magsår Läkartidningen 2017; 114: ECUW. 28267193

[10] Black CJ Paine PA Agrawal A , et al. British Society of Gastroenterology guidelines on the management of functional dyspepsia. Gut 2022; 71(9): 1697–1723. 35798375 10.1136/gutjnl-2022-327737 PMC9380508

[11] Moayyedi P Lacy BE Andrews CN , et al. ACG and CAG clinical guideline: management of dyspepsia. Am J Gastroenterol 2017; 112(7): 988–1013. 28631728 10.1038/ajg.2017.154

[12] Ford AC Yuan Y Moayyedi P. *Helicobacter pylori* eradication therapy to prevent gastric cancer: systematic review and meta-analysis. Gut 2020; 69(12): 2113–2121. 32205420 10.1136/gutjnl-2020-320839

[13] Räsänen N van Nieuwenhoven M. Gastroscopy in younger patients: an analysis of referrals and pathologies. Eur J Gastroenterol Hepatol 2021; 33(10): 1266–1273. 34334711 10.1097/MEG.0000000000002260

[14] de Jong JJ Lantinga MA Thijs IME , et al. Systematic review with meta-analysis: age-related malignancy detection rates at upper gastrointestinal endoscopy. Therap Adv Gastroenterol 2020; 13: 1756284820959225. 33209123 10.1177/1756284820959225 PMC7645776

[15] Von Elm E Altman DG Egger M , et al.; STROBE Initiative. The Strengthening the Reporting of Observational Studies in Epidemiology (STROBE) statement: guidelines for reporting observational studies. Ann Intern Med 2007; 147(8): 573–577. 17938396 10.7326/0003-4819-147-8-200710160-00010

[16] Rajan S Amaranathan A Lakshminarayanan S , et al. Appropriateness of American Society for Gastrointestinal Endoscopy Guidelines for upper gastrointestinal endoscopy: a prospective analytical study. Cureus 2019; 11(2): e4062. 10.7759/cureus.4062 PMC6464286 31016089

[17] Lanas A Chan FKL . Peptic ulcer disease. Lancet 2017; 390(10094): 613–624. 28242110 10.1016/S0140-6736(16)32404-7

[18] Expert Panel on Gastrointestinal Imaging; Vij A Zaheer A Kamel IR , et al. ACR appropriateness criteria(R) epigastric pain. J Am Coll Radiol 2021; 18(11S): S330–S339. 10.1016/j.jacr.2021.08.006 34794592

[19] Kanold P Nyhlin N Szabo E , et al. The Swedish standardized course of care-diagnostic efficacy in esophageal and gastric cancer. Diagnostics 2023; 13(23): 3577. 38066818 10.3390/diagnostics13233577 PMC10706212

[20] Fallone CA Chiba N van Zanten SV , et al. The Toronto consensus for the treatment of *Helicobacter pylori* infection in adults. Gastroenterology 2016; 151(1): 51–69.e14. 10.1053/j.gastro.2016.04.006 27102658

[21] Gupta K Groudan K Jobbins K , et al. Single-center review of appropriateness and utilization of upper endoscopy in dyspepsia in the United States. Gastroenterol Res 2021; 14(2): 81–86. 10.14740/gr1370 PMC8110238 34007349

[22] Buri L Zullo A Hassan C , et al. Upper GI endoscopy in elderly patients: predictive factors of relevant endoscopic findings. Intern Emerg Med 2013; 8(2): 141–146. 21538157 10.1007/s11739-011-0598-3

[23] Standardiserade Vårdförlopp i Cancervården—Lägesrapport 2015. Socialstyrelsen 2015, https://www.socialstyrelsen.se/globalassets/sharepoint-dokument/artikelkatalog/ovrigt/2015-11-6.pdf (accessed 19 April 2024).

[24] Rasmussen S Haastrup PF Balasubramaniam K , et al. Predictive values of upper gastrointestinal cancer alarm symptoms in the general population: a nationwide cohort study. BMC Cancer 2018; 18(1): 440. 29669540 10.1186/s12885-018-4376-8 PMC5907174

[25] Vakil N Moayyedi P Fennerty MB , et al. Limited value of alarm features in the diagnosis of upper gastrointestinal malignancy: systematic review and meta-analysis. Gastroenterology 2006; 131(2): 390–401; quiz 659–660. 10.1053/j.gastro.2006.04.029 16890592

